# Clinical and radiographic variables related to implants with simultaneous grafts among type 2 diabetic patients treated with different hypoglycemic medications: a retrospective study

**DOI:** 10.1186/s12903-021-01583-3

**Published:** 2021-04-27

**Authors:** Shaojie Shi, Feng Ding, Xiangdong Liu, Lei Wang, Xingxing Wang, Sijia Zhang, Guoqiang Zhao, Yingliang Song

**Affiliations:** State key Laboratory of military Stomatology and National Clinical Research Center for Oral Diseases and Shaanxi Engineering Research Center for Dental Materials and Advanced Manufacture, Department of Oral Implants, School of Stomatology, The Fourth Military Medical University, Xi’an, 710032 Shaanxi China

**Keywords:** Type 2 diabetic mellitus, Dental implant, Marginal bone loss, Hypoglycemic agents, Guided bone regeneration

## Abstract

**Background:**

The influence of different hypoglycemic agents on peri-implant variables among type 2 diabetes mellitus patients is still unclear. Therefore, the aim of this study was to assess the radiographic marginal bone loss and clinical parameters around implants in patients using different hypoglycemic agents.

**Methods:**

In this retrospective cohort study, the dental implant records of type 2 diabetes mellitus patients who met the inclusion criteria were collected. The patients using only single medication as follows: insulin, metformin, or glucagon-like peptide-1 (GLP-1) drugs, were grouped according to their medication. These patients received implant placement with the same initial status, and all the prosthesis restorations were cement-retained ceramic crowns. The peri-implant marginal bone levels were evaluated by periapical radiographs immediately after implant placement and at 1 and 2-year follow-up visits. The baseline characteristics were compared among groups. The peri-implant radiographic marginal bone loss and clinical parameters were preliminarily compared using the Kruskal–Wallis test, and then the covariates were controlled by covariance analysis. Bonferroni post hoc adjustment test was performed for the multiple comparisons.

**Results:**

After a review of more than 7000 medical records, a total of 150 patients with 308 implants at 1-year follow-up were assessed. The peri-implant marginal bone loss in the GLP-1 drug group was significantly smaller than the insulin group and metformin group (*P* < 0.01). The radiographic bone loss in the metformin group was higher than the insulin group (*P* < 0.05). Some of these included patients were lost to follow-up. Only 74 patients with 129 implants completed the 2-year follow-up. The radiographic bone loss in the metformin group was still higher than the insulin group (*P* < 0.05) and GLP-1 group (*P* < 0.01). There was no significant difference in the BOP (+) and the mean PD among groups (*P* > 0.05).

**Conclusions:**

The radiographic variables were not exactly the same among the patients with different hypoglycemic agents at both the 1 and 2-year follow-ups. After ensuring consistency in baseline characteristics, the positive effect of GLP-1 drugs on peri-implant bone remodeling may be no less than insulin or metformin. More studies are needed to verify the direct effect of these drugs on peri-implant bone.

*Clinical trial registration number* ChiCTR2000034211 (retrospectively registered).

## Background

A large number of individuals have been diagnosed with type 2 diabetes mellitus (T2DM), and a recent report in 2019 showed that, the prevalence of T2DM worldwide reached 8% in 2018 [1]. Due to the relationship between T2DM and periodontitis [2], there is a large proportion of patients with missing teeth in the T2DM population. Implant-supported denture restoration has been an excellent treatment for the loss of teeth and T2DM individuals have a large requirement for dental implant treatment. However, T2DM patients often face the challenge of atrophic alveolar bone width at implant sites [3]. The minor and moderate atrophic ridge often requires horizontal bone augmentation by guided bone regeneration (GBR)with the combination of bone graft materials and barrier membranes [4]. It is reported that the hyperglycemia impacts the clinical effect of dental implants [5]. Some studies have shown that the early osseointegration around implants would be compromised by T2DM even if glucose is strictly controlled [6]. Thus, it is crucial for clinicians to improve the efficacy of implant treatment in T2DM patients.

Dental implant treatment success is related to the osseointegration and peri-implant bone remodeling around implants [7, 8]. Previous research has shown that diabetes impacts bone remodeling around implants, especially at the early stage of osseointegration [9]. Several factors would influence the bone remodeling around dental implants, including the blood glucose and physical illnesses of patients, the position of fixture and the major surface treatment of the implants [10]. The marginal bone loss (MBL) in diabetes patients is greater than that in nondiabetic individuals, regardless of whether the former group is glycemic controlled [11]. The higher MBL in T2DM patients could show the impaired bone condition around implants. Therefore, MBL was considered an important radiographic parameter for bone remodeling around implants in the present study.

Recently, abundant evidence has revealed the role of hypoglycemic agents in bone metabolism [12]. There is growing concern regarding their direct bone targeting effect. For example, insulin and metformin could be beneficial to bone tissue [13], and may improve the outcome of dental implants. It was reported that local application of insulin could promote osseointegration in T2DM rats [14]. Glucagon-like peptide-1 (GLP-1) drugs have the effect of controlling blood glucose and regulating bone metabolism as hypoglycemic agents [15], so GLP-1 drugs might have the potential to promote osseointegration around implants. Currently, there is no clinical evidence of hypoglycemic drugs interfering with bone remodeling around implants. Therefore, the present study aimed to obtain more detailed information about the clinical and radiographic variables around implants in T2DM patients using different hypoglycemic agents.

## Methods

### Ethical protocol

This retrospective cohort study was approved by the Ethics Committee of School of Stomatology, the Fourth Military Medical University (Ethics Approval Number: IRB-REV-2020045), and was in compliance with the Helsinki Declaration. All patients were given complete information about the treatment and signed informed consent forms before surgery. The implant treatment was performed by the same doctor and the trauma was minimized. This study is reported in accordance with the STROBE (Strengthening the Reporting of Observational Studies in Epidemiology) statement.

### Study design and setting

This retrospective cohort study was designed to compare the clinical and radiographic variables related to implants with simultaneous GBR in T2DM patients using different hypoglycemic agents. The examiners collected dental records of T2DM patients who underwent implant surgery from January 2015 to November 2019 at the hospital. After information collection, one examiner divided them into three groups based on their major hypoglycemic agent species: insulin, metformin, or GLP-1 drugs. The status immediately after surgery was recorded as baseline. The marginal bone loss (MBL) at the 1 and 2-year follow-ups after implant placement were respectively measured to assess the bone remodeling around implants. The clinical inflammatory parameters at the 2-year follow-up, including bleeding on probing (BOP) and probing depth (PD), were collected. To ensure the principle of blinding, the groups were concealed and another independent examiner assessed the data among groups. The sample size calculation was performed using the PASS software. The value of 80% (Beta = 0.20) was used for power and the value of 0.05 has been used for significance level. Specify the values of the minimum detectable difference as 0.11 mm and the standard deviation within a group is set as 0.1. There are three groups in this research and the sample size of implants in each group required is 43. The researchers reviewed and collected all the patient records eligible for the inclusion criteria and the sample size in each group was larger than the reference value.

### Eligibility criteria

The inclusion criteria were as follows: (a) medically diagnosed T2DM; (b) good blood glucose control before surgery (HbA1c% ≤ 8% [16]); (c) treatment with only one of these hypoglycemic agents: metformin, insulin or GLP-1 drugs; (d) implant site with Seibert class I ridge deficiencies resulting in implant thread exposure, which was treated only with GBR (graft mass ≤ 0.25 g); (e)good oral hygiene care with regular semidiurnal brushing and semiannual professional cleaning; and (f) 40 to 70 years old.

The exclusion criteria were as follows: (a) poor glycemic control (HbA1c% > 8%) or severe diabetic complications; (b) osteoporosis; (c) use of bisphosphonates or steroids within three years before the implant placement; (d) use of two or more hypoglycemic drugs; (e) with any tobacco use or alcohol consumption; and (f) uncontrolled periodontitis before treatment or recurrence of periodontitis at follow-up visits.

### Information collection

The patient records were reviewed, and the baseline information on the patients and implants was obtained by two examiners. Another researcher was responsible for supervising the operation and resolving any disagreements. The patient information included age, sex, HbA1c, medication, bone augmentation and oral hygiene care. The implant information included the size, location, arch, the position of fixture and major surface treatment of implants. The surgical complications after implant placement were recorded, including wound bleeding, swelling and membrane exposure. Data on infections around implants were also obtained. The patients with any missing information were excluded before analyzing the baseline formation and no missing data was detected in the present study.

### Radiographic analysis at the 1 and 2-year follow-ups after implant placement

**Fig. 1:**
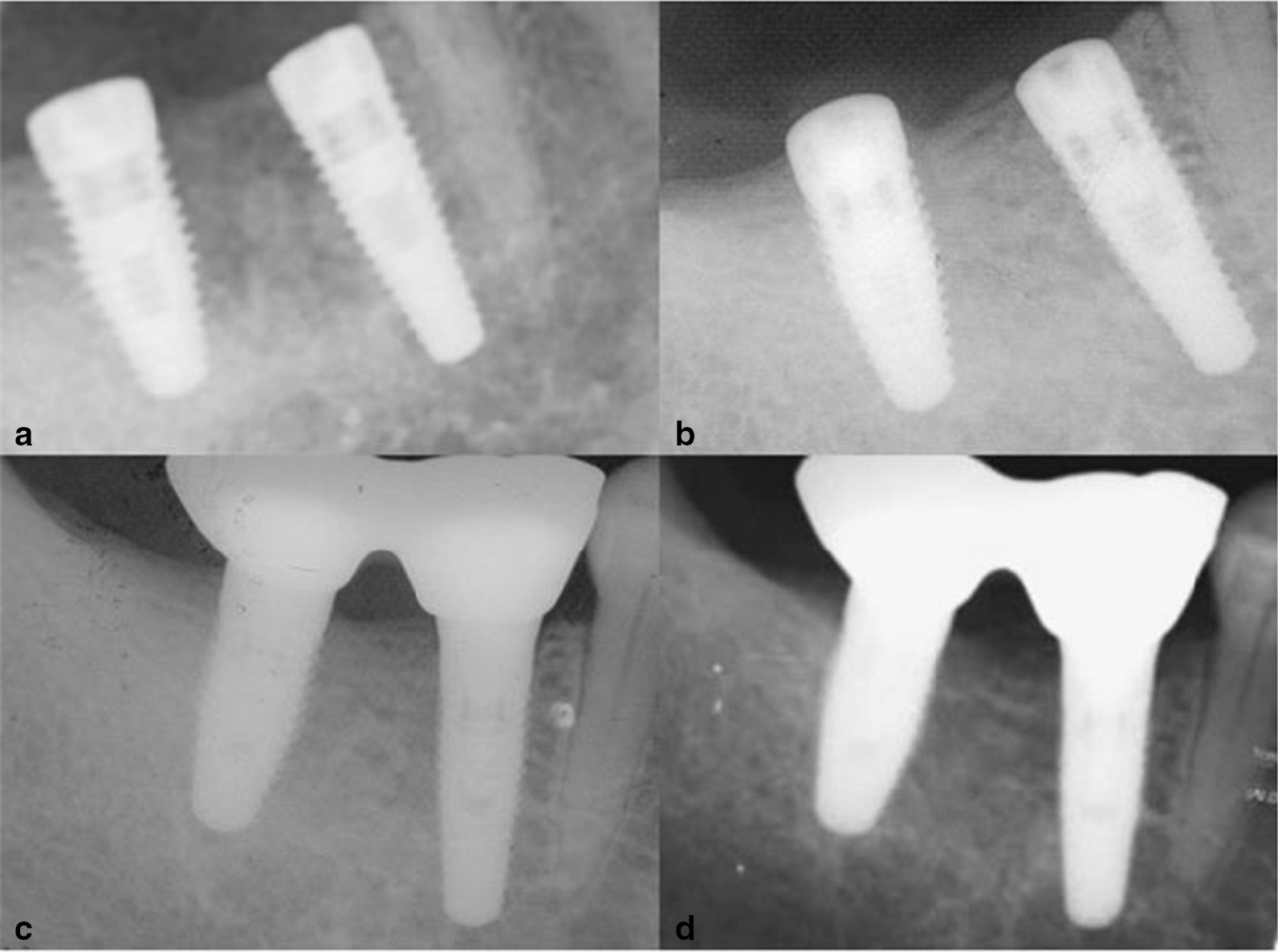
Periapical radiographs taken for implants in the same patient. **a** Immediately after the implant placement. **b** Before prosthetic installation. **c** Immediately after prosthetic installation(at the 1-year follow-up after the implant placement). **d** At the 2-year follow-up after the implant placement

After gathering the information on patients and implants, the radiographic materials were collected immediately after implant placement and at follow-up visits. All radiographs collected were viewed on a computer screen using Digimizer v5.4.5 (MedCalc Inc. Mariakerke, Belgium) software. A standardized digital dental periapical radiographic evaluation performed immediately after implant placement was recorded as the baseline. Then, the radiographic evaluations carried out at the 1 and 2-year follow-ups after implant placement, were respectively recorded (Fig. 1).

**Fig. 2:**
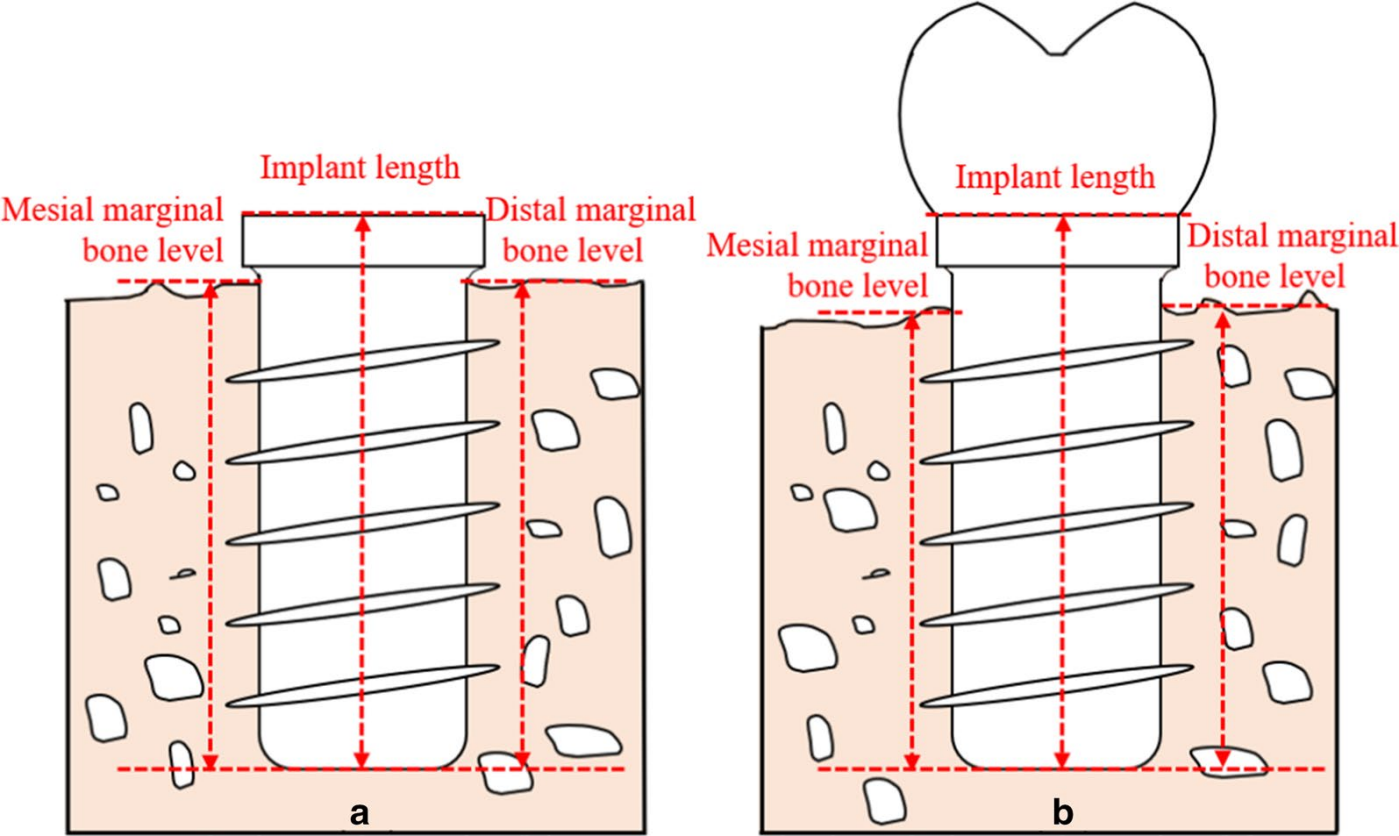
The method of measuring the marginal bone levels. **a** The marginal bone levels immediately after implant placement; **b** the marginal bone levels at the follow-ups

All radiographic measurement was performed by a professional radiographic examiner and the Kendall value for intra-examiner reliability was 0.889. The radiographic examiner measured the marginal bone levels, defined as the vertical distance from the tip of the implant body to the coronal edge of the first bone-to-implant contact (Fig. 2). The mesial and distal marginal bone levels of the same implant were measured. The image error of magnification was calibrated by the reference value of the actual implant length. The MBL was calculated as the change in marginal bone levels from immediately after implant placement to the different follow-up time points. And the MBL in percentage was calculated as the percentage of MBL in contrast to the implant length.

Method for measuring marginal bone levels:

- Actual marginal bone levels = measured radiographic marginal bone levels × actual length of implant/measured radiographic length of implant.
- Method for calculating marginal bone loss:
- MBL = marginal bone levels immediately after implant placement—marginal bone levels at follow-up.

### Clinical parameters at the 2-year follow-up after implant placement

The clinical parameters recordings at the 2-year follow-up after implant placement were collected and analyzed, including peri-implant BOP and PD. These clinical parameters were measured at six sites around the implants, including mesiobuccal, midbuccal, distobuccal, distolingual/ distopalatal, midlingual/ midpalatal, and mesiolingual/ mesiopalatal. The BOP was recorded as (+) in the patient records when the bleeding after probing appeared at one or more of the six sites. And the PD values of the six sites recorded in the patient records were calculated for the mean PD.

### Statistical analysis

Statistical analysis was performed using specialized software (SPSS v25.0, IBM, Chicago, Illinois). The baseline information on the patients and implants was analyzed by the chi-square test or analysis of variance. After analyzing the baseline information, the dental implant was considered as the unit of analysis of the present study. Dependent variables were evaluated for a normal distribution by the Shapiro–Wilk test. The data are expressed as the means and SDs. Peri-implant MBL and clinical parameters among groups were preliminarily compared using the Kruskal–Wallis test. Bonferroni post hoc adjustment test was performed for the multiple comparisons. The different baseline variables among groups were considered as covariates. Covariance analysis was performed to make the different baseline variables consistent among groups and then compare the radiographic and clinical parameters among groups. Bonferroni post hoc adjustment test was performed for the multiple comparisons after controlling the covariances. The statistical significance threshold was set at *P* < 0.05.

## Results

### The general condition of the patients and implants

**Fig. 3:**
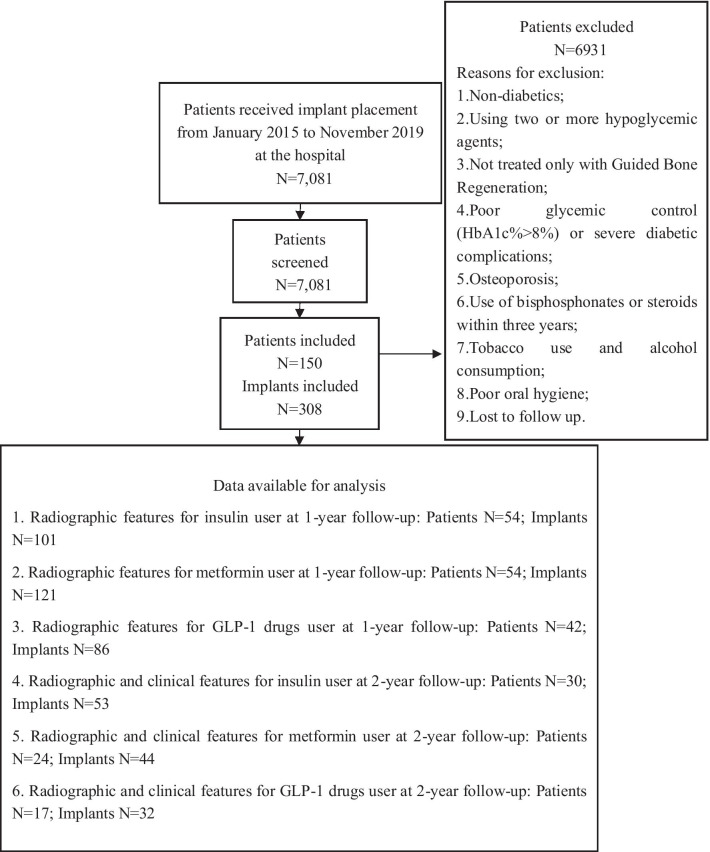
The flowchart as recommended by the STROBE

After a review of 7,081 medical records, a total of 150 patients with 308 implants with GBR completing a 1-year follow-up after implant placement were eligible for this study. Among these patients, only 71 patients with 129 implants completed a 2-year follow-up after implant placement, and the others lost to follow-up were excluded. The reasons for this low response rate at the 2-year follow-up may be complicated, and include the high cost for patients to follow-up, the low compliance and a lack of awareness of patients to follow up regularly. The analysis at the 1 and 2-year follow-ups were performed respectively. The system of implant included Straumann, Basel, Switzerland and Nobel Biocare, Gothenburg, Sweden. The implant condition was analyzed by the position of fixture and the major surface treatment to avoid conflicts of interest. These patients received implant placement with the same initial status and all the prosthesis restorations were cement-retained ceramic crowns. Sixty-one implants in groups with GBR had experienced surgical complications but no implants suffered infection. No further prosthesis complications were reported during the follow-up period. Flow-chart resuming the patient selection process, showing causes for exclusion and the number of patients and implants with available data for the main analysis presented in the study (Fig. 3).

The basic information on patients and implants with GBR at the 1 and 2-year follow-ups is shown in Tables 1 and 2, respectively. At the 1-year follow-up, there were 54 patients and 101 implants in the insulin group, 54 patients and 121 implants in the metformin group, 42 patients and 86 implants in the GLP-1 drug group. These groups were comparable with respect to age, HbA1c, sex, implant location and the major surface treatment of implants (*P* > 0.05). However, the position of fixture was significantly different among the groups (*P* < 0.01).

**Table 1:** Information on the patients and implants at the 1-year follow-up after implant placement

Variable	Insulin group	Metformin group	GLP-1 drug group	*P*
Total patients (n)	54	54	42	
Age (years)	56.5 ± 9.4	55.5 ± 8.2	56.8 ± 8.7	0.726
HbA1c (%)	7.0 ± 0.5	6.9 ± 0.5	7.0 ± 0.5	0.714
*Sex*				
Male/female	40/14	46/8	33/9	0.358
Total implants (n)	101	121	86	
Length (mm)	10.5 ± 1.5	10.8 ± 1.7	10.8 ± 1.9	0.392
*Arch*				
Maxillary/mandibular	61/40	72/49	44/42	0.376
*Location*				
Anterior/posterior	22/79	34/87	25/61	0.448
*Position of fixture*				
Submerged /non-submerged	54/47	55/66	61/25	0.001**
*Major surface treatment*				
SLA/Anodic oxidation	54/47	73/4	39/47	0.103
*Restoration type*				
Single/multiple ceramic restoration	39/62	38/83	35/51	0.332

The significance of bold: The position of fixture among groups is significantly different among groups (*P* < 0.01). **p < 0.01

**Table 2:** Information on the patients and implants at the 2-year follow-up after implant placement

Variable	Insulin group	Metformin group	GLP-1 drug group	*P*
Total patients (n)	30	24	17	
Age (y)	55.9 ± 8.9	55.0 ± 8.0	55.5 ± 7.7	0.926
HbA1c (%)	7.1 ± 0.5	7.4 ± 0.3	7.3 ± 0.5	0.188
*Sex*				
Male/female	20/10	21/3	12/5	0.197
Total implants (n)	53	44	32	
Length (mm)	10.6 ± 1.7	10.8 ± 1.4	11.0 ± 1.9	0.568
*Arch*				
Maxillary/mandibular	33/20	23/21	20/12	0.544
*Location*				
Anterior/posterior	10/43	12/32	6/26	0.544
*Position of fixture*				
Submerged /non-submerged	33/20	21/23	22/10	0.150
*Major surface treatment*				
SLA/Anodic oxidation	23/30	28/16	14/18	0.096
*Restoration type*				
Single/multiple ceramic restoration	25/28	10/34	13/19	0.042*

The significance of bold: Restoration type among groups is significantly different among groups (*P* < 0.05). *p < 0.05

A total of 71 patients with 129 implants completed a 2-year follow-up(30 patients and 53 implants in the insulin group, 24 patients and 44 implants in the metformin group, 17 patients and 32 implants in the GLP-1 drug group). The most of the baseline information of patients and implants among the groups were comparable, including age, HbA1c, sex, location, the position of fixture and the major surface treatment of implants (*P* > 0.05), while the restoration type was significantly different among the groups (*P* < 0.05).

### A preliminary comparison of radiographic and clinical parameters of implants among the groups at the 1 and 2-year follow-ups after implant placement

The results of the Shapiro–Wilk test showed that not all indexes fit the normal distribution of data, so the preliminary comparison of MBL among groups was performed by the Kruskal–Wallis test. The results showed that the MBL was not the same among these medication groups. At the 1-year follow-up, the mesial and distal MBL in the GLP-1 drug group (mesial: 0.38 ± 0.12 mm, distal: 0.36 ± 0.12 mm) were smaller than the metformin group (mesial: 0.45 ± 0.14 mm (*P* < 0.01), distal: 0.47 ± 0.13 mm (*P* < 0.01)) and insulin group (mesial: 0.43 ± 0.14 mm (*P* < 0.05), distal: 0.42 ± 0.13 mm (*P* < 0.01)). And the distal MBL in the metformin group (0.47 ± 0.13 mm) was higher than that in the insulin group (0.42 ± 0.13 mm) (*P* < 0.05). Similarly, the mesial MBL in percentage to the implant length in metformin group (4.30 ± 1.58%) was significantly higher than GLP-1 drug group (3.67 ± 1.50%) (*P* < 0.01). The distal MBL in percentage to the implant length in the GLP-1 drug group (3.42 ± 1.38%) were smaller than the metformin group (4.45 ± 1.56%) (*P* < 0.01) and insulin group (4.04 ± 1.25%) (*P* < 0.01).

At the 2-year follow-up, the mesial and distal MBL parameters in the insulin group (mesial: 0.63 ± 0.15 mm, distal: 0.61 ± 0.17 mm) (*P* < 0.05) were smaller than those in the metformin group (mesial: 0.72 ± 0.14 mm, distal: 0.68 ± 0.13 mm). And the mesial MBL in the GLP-1 drug group (0.58 ± 0.13 mm) (*P* < 0.01) was smaller than the metformin group. Additionally, the mesial MBL in percentage to the implant length in the metformin group (6.83 ± 1.73%) was higher than that in GLP-1 drug group (5.48 ± 1.48%) (*P* < 0.01). Regarding the clinical parameters at the 2-year follow-up, there was no significant difference in the BOP (+) (*P* > 0.05), and the mean PD in the insulin group (1.30 ± 0.24 mm) was comparable to that in the metformin group (1.37 ± 0.24 mm) and GLP-1 drug group (1.27 ± 0.31 mm) (*P* > 0.05).

### Regression analysis to control the position of fixture and compare the MBL among groups at the 1-year follow-up

The difference in MBL among groups was preliminarily stated by the results of the Kruskal–Wallis test. However, the position of fixture of implant was significantly different among groups, and this difference would influence the comparison of MBL as the covariate among groups. The position of fixture of implants among groups at the 1-year follow-up should be controlled as confounders by regression analysis. The mesial and distal MBL at the 1-year follow-up still showed significant differences among the groups even after controlling for the position of fixture (*P* < 0.05). Table 3 shows that the mesial and distal MBL in the metformin group (*P* < 0.01) was higher than the GLP-1 drug group at the 1-year follow-up. The distal MBL in the insulin group was smaller than that in the metformin group (*P* < 0.05) and higher than GLP-1 drug group (*P* < 0.01) after controlling for consistency in the position of fixture among groups.

**Table 3:** The comparison of the MBL among groups at the 1-year follow-up after controlling the position of fixture as a covariate

MBL	Insulin group	Metformin group	GLP-1 drug group
Mesial MBL in millimeters	0.42 ± 0.13	0.45 ± 0.12^a^	0.38 ± 0.14
Distal MBL in millimeters	0.42 ± 0.13^a,b^	0.46 ± 0.12^a^	0.36 ± 0.14
Mesial MBL in percentage	4.07 ± 0.14	4.23 ± 0.13	3.79 ± 0.12
Distal MBL in percentage	4.02 ± 0.14	4.38 ± 0.13^a^	3.54 ± 0.15

^a^Compared with GLP-1 drug group (*P* < 0.01)

^b^Compared with Metformin group (*P* < 0.05)

### Regression analysis to control the restoration type and compare the parameters among the groups at the 2-year follow-up

The restoration type of implants among the groups at the 2-year follow-up should be controlled as confounders by regression analysis (Table 4). The mesial and distal MBL parameters in the insulin group were smaller than those in metformin group (*P* < 0.05). And the mesial MBL in the GLP-1 drug group was smaller than metformin group (*P* < 0.01). Additionally, the mesial MBL in percentage to the implant length in the metformin group was higher than that in the GLP-1 drug group (*P* < 0.01). Regarding the clinical parameters at the 2-year follow-up, there was no significant difference in the BOP (+) (*P* > 0.05) and the mean PD (*P* > 0.05) among groups.

**Table 4:** The comparison of the MBL and clinical parameters among groups at the 2-year follow-up controlling the restoration type as a covariate

MBL	Insulin group	Metformin group	GLP-1 drug group
Mesial MBL in millimeters	0.63 ± 0.02^c^	0.72 ± 0.02	0.58 ± 0.03^d^
Distal MBL in millimeters	0.60 ± 0.02^c^	0.69 ± 0.03	0.61 ± 0.03
Mesial MBL in percentage	6.04 ± 0.22	6.83 ± 0.24	5.47 ± 0.28^d^
Distal MBL in percentage	5.81 ± 0.25	6.58 ± 0.28	5.66 ± 0.32
BOP (+)	44 (83.0%)	36 (81.8%)	27 (84.3%)
PD (mm)	1.30 ± 0.04	1.38 ± 0.04	1.27 ± 0.05

^c^Compared with Metformin group (*P* < 0.05)

^d^Compared with Metformin group (*P* < 0.01)

## Discussion

The influence of diabetes on the properties of individual bone impacts the outcomes of dental implants. In cases where diabetes cannot be completely cured, clinicians generally use hypoglycemic drugs to reduce the adverse effects of diabetes. Numerous studies have reported the direct effect of hypoglycemic drugs on systemic bone metabolism [17–19]. However, little attention has been paid to the effect of hypoglycemic drugs on peri-implant bone. In the present study, the peri-implant radiographic parameters showed different characteristics among patients using different hypoglycemic agents. The results could serve as a reference for the effect of drugs on systemic bone.

The MBL around implants could show the bone healing and remodeling process, and regarding the definition of a successful implant, Albrektsson (1985) declared that the MBL at the first year should be no more than 2 mm and less than 0.2 mm annually in subsequent years [20].This study showed that the MBL was not exactly the same among groups. The MBL parameters in the insulin group and metformin group were higher than the GLP-1 group at the 1-year follow-up. The MBL in the metformin group was higher than that in the insulin group and GLP-1 drug group at the 2-year follow-up. During these periods, the bone and bone substitution material underwent the bone healing and remodeling process, and this process could be influenced by the bone microenvironment around the implants. The different MBL among groups showed the possibility of these drugs interfering with bone remodeling around the implant in T2DM patients in different manners. Based on these results, the hypoglycemic agents have the potential to influence the bone healing and remodeling around an implant at the early stage, and their potentials may be different. There were different results for the 1-year and 2-years of follow-up. At the 1-year follow-up, the MBL in insulin was higher than GLP-1 drug group, but there was no statistically significant difference in MBL between insulin and GLP-1 drugs at 2-year follow-up. The reason for the different results might be complicated. The first explanation might be the effect of these drugs on peri-implant bone remodeling varied with healing time. The other explanation might be that the sample size was different between the 1-year and 2-year follow-ups. This controversy should be further researched in the future study.

In addition, the present results showed that there was no significant difference in BOP ( +) and PD values among the groups. Although diabetes could be related to the periodontal inflammation [21], the relationship between peri-implant inflammation and hypoglycemic medication is still uncertain. Unexpectedly, some hypoglycemic agents can influence peri-implant inflammation, and it had been reported that the metformin in gel could even promote the benefit of mechanical periodontal therapy and relieve the inflammatory burden in patients with chronic periodontitis [22]. This study showed that the BOP (+) and PD in the GLP-1 drug group was no more than the metformin group. This comparison in peri-implant inflammation parameters among hypoglycemic agents may demonstrate the positive effect of the GLP-1 drugs on peri-implant hard and soft tissue.

The results showed that GLP-1 drugs may have a more positive effect on the peri-implant bone compared with classic hypoglycemic agents. GLP-1 is known as an incretin hormone regulating blood glucose by promoting insulin release and the GLP-1 may influence bone metabolism involving the inhibition of osteoclasts and activation of osteoblasts [23]. Preclinical studies have reported that GLP-1 receptors are widely distributed, and knockout of the GLP-1 receptor gene would lead to severe bone changes related to the abnormality of osteoclasts [24]. Additionally, the GLP-1 could be beneficial to the metabolism, including the provision of protection for the cardiovascular and nervous systems as an antioxidant [25]. GLP-1 may have the potential to protect the vascular function from high-glucose-induced oxidative injury, which could improve bone formation in T2DM individuals [26].

Several GLP-1 receptor agonists have been developed for the treatment due to the beneficial effect of GLP-1 on metabolism, such as liraglutide, exendin-4 and exenatide [27]. It has been reported that GLP-1 receptor agonists could significantly reduce the risk of fracture than other hypoglycemic drugs [28]. Another meta-analysis provided evidence that exenatide may have advantages in preventing fracture risk over other hypoglycemic drugs [29]. Liraglutide could have a beneficial effect on bone health by increasing the serum osteocalcin and decreasing the serum c-terminal telopeptide of type 1 collagen (a bone resorption marker) [30]. Exendin-4 could inhibit osteoclast formation and the expression of TNF-α in macrophages to prevent bone resorption [31]. Exenatide could improve the trabecular bone mass and protect the development of the skeletal against T2DM [32]. Exenatide could also improve factors beneficial to osseointegration around implants in a T2DM rat model [33]. However, the results showed no difference in MBL between the insulin and GLP-1 drug groups at the 2-year follow-up. A previous study also showed that the influence of these two agents on bone around implants may be similar. Specifically, Li [34] indicated that there was no difference in the effects of exenatide and insulin on bone turnover markers and bone mineral density, but the observation time was short. More studies are needed to clarify the specific effects of GLP-1 receptor agonist on bone tissue compared with insulin.

Metformin and insulin are both classic hypoglycemic medications and there are many studies about their positive influence on bone tissue [35, 36]. The results showed that the distal MBL in the metformin group was higher than that in the insulin group at the 1 and 2-year follow-ups. There are still competing ideas regarding the comparison of the bone metabolism effect between these two drugs. Raj [37] noted that compared with metformin, insulin could significantly protect bone through osteocalcin and other pathways. However, Hidayat [38] determined that metformin could reduce the fracture risk and protect bone metabolism compared with insulin. The underlying mechanism of this contradiction is unclear. The explanation may be that diabetes is generally more severe in insulin users than oral medicine users and the fracture risk cannot accurately reflect the influence of drugs on bone metabolism. This study selected patients with good blood glucose control and no severe complications. The interference of different conditions was removed to the extent possible to increase the reliability of the results.

Several studies have demonstrated the positive effect of insulin on bone tissue [39]. Insulin has a positive effect by ameliorating the dramatic impact in bone mineral density and bone microstructure induced by diabetes mellitus [40]. It has been shown that insulin could promote bone formation and prevent diabetes-induced bone loss by upregulating the serum osteogenesis factor, including osteoprotegerin (OPG) and osteocalcin (OC) [35]. The OC would in turn regulate the glucose homeostasis by promoting the expression of insulin, and a positive feedforward loops was established between bone metabolism and insulin [41]. Some studies have shown that a local injection of insulin can promote early fracture healing in diabetic animals [42]. Moreover, local or systematic use of insulin could improve implant osseointegration in osteoporotic and diabetic rats [43, 44]. The dental implants could even serve as a novel route of insulin delivery by an implant-mediated drug delivery system [45]. Another study has shown that insulin could promote angiogenesis [46], which is also a conducive factor to early osseointegration.

Metformin is a first-line drug for diabetes treatment and has a positive effect to promote osteogenesis and inhibit bone resorption [47]. T2DM individuals could have a higher marrow fat content than nondiabetic individuals, while metformin could reduce the marrow adiposity in T2DM [48]. The low concentrations of metformin could alleviate the impact of oxidative stress on periodontal ligament stem cells (PDLSCs), facilitate the osteogenic differentiation, inhibit adipose differentiation of PDLSCs and then promote alveolar bone regeneration for the periodontitis treatment [36]. The positive effects of metformin on microangiopathy and inflammation could also be found in bone formation and mineral anabolism [49]. However, there are some studies reporting the side effect of metformin on bone. A post hoc study reported that metformin decreased the level of bone turnover factors and hindered the bone remodeling in polycystic ovary syndrome [50]. This effect may compromise the self-repair of minor bone tissue damage. In this study, the MBL in the insulin group was smaller than that in the metformin group, which may be related to their effect on bone metabolism. The explanation for the conflicting effect of metformin on bone metabolism might be that metformin would act on bone in a dose dependent manner [51].

Currently, the major controversy regarding the bone targeting effect of some hypoglycemic drugs entails their indirect effect on bone by controlling blood glucose [52]. Firstly, it is noteworthy that all T2DM patients included in this study had good controlled blood glucose, with HbA1c less than 8%. Additionally, the results showed that MBL was different among groups, even though the patients in each group reported good controlled blood glucose. The results meant that these drugs may have different influences on peri-implant bone unrelated to the hypoglycemic effect. The process of bone remodeling around implants requires an environment conducive to healthy bone metabolism, but the diabetes would impact the bone formation and bone resorption [53]. Therefore, T2DM patients undergoing implant surgery should receive hypoglycemic drugs beneficial to bone metabolism, such as insulin and GLP-1 drugs. However, this adjustment of the medication plan should not violate the clinical principle of diabetes treatment. Hence, the results of this study are more useful for patients who have flexibility regarding agent use. One limitation of this retrospective study is that blood glucose could not be monitored in real time, and the basic diabetes among patients may not be precisely the same. Therefore, further laboratory-based investigations should be executed to guarantee blood glucose control and basic diabetes more strictly to eliminate bias in future studies.

Another contribution of the present study is that it provides feedback on clinical systemic bone research. Previous research on the systematic bone target effect of hypoglycemic drugs focused more on the fracture risk, bone mineral density and bone-related biochemical indexes [54]. However, these indexes could not accurately reflect the influence of drugs on bone. The bone mineral density in patients with T2DM may be higher than that in normal individuals, and the fracture risk in T2DM patients could be high [55]. The high fracture risk may be related to damaged bone quality, a higher incidence of falls and severe complications of diabetes. While the MBL around the implant is an important criterion for the efficacy of implant treatment and could be affected by bone metabolism [56]. Therefore, MBL could serve as an indicator for the effect of hypoglycemic agents on bone remodeling around implants. This study focused on the MBL around implants with GBR in different hypoglycemic agent groups. However, it is beyond the scope of this study to examine the horizontal change in the alveolar ridge at the implant site as this study was based on two-dimensional images. Follow-up studies on the horizontal change in the ridge should be executed using cone beam computed tomography.

## Conclusion

According to the results, different hypoglycemic agents may have different influences on bone remodeling around implants. Compared with the classic hypoglycemic medication, the positive role of GLP-1 drugs in bone remodeling around implants is not weaker. As an incretin with both hypoglycemic and bone metabolism effects, GLP-1 is a potential target for improving the clinical effect of dental implants in T2DM patients. However, the present study could not provide a high level of evidence regarding the influence of these drugs on the bone condition around implants. Because the skeletal effects of hypoglycemic drugs are very complex, more clinical evidence is still needed through future research.

## Data Availability

The data generated and analyzed during this study are available from corresponding author on reasonable request.

## Abbreviations

T2DM: Type 2 diabetic mellitus
GLP-1: Glucagon-like peptide-1
MBL: Marginal bone loss
GBR: Guided bone regeneration
BOP: Bleeding on probing
PD: Probing depth
